# Engineering pH and Temperature-Triggered Drug Release with Metal-Organic Frameworks and Fatty Acids

**DOI:** 10.3390/molecules29225291

**Published:** 2024-11-08

**Authors:** Wanying Wei, Ping Lu

**Affiliations:** Department of Chemistry and Biochemistry, Rowan University, Glassboro, NJ 08028, USA; weiw8@rowan.edu

**Keywords:** zeolitic imidazolate framework-8, coaxial electrospray, phase-change material, stimuli-responsive drug delivery, core-shell microparticles

## Abstract

This study reports the successful synthesis of core-shell microparticles utilizing coaxial electrospray techniques, with zeolitic imidazolate framework-8 (ZIF-8) encapsulating rhodamine B (RhB) in the core and a phase change material (PCM) shell composed of a eutectic mixture of lauric acid (LA) and stearic acid (SA). ZIF-8 is well-recognized for its pH-responsive degradation and biocompatibility, making it an ideal candidate for targeted drug delivery. The LA-SA PCM mixture, with a melting point near physiological temperature (39 °C), enables temperature-triggered drug release, enhancing therapeutic precision. The structural properties of the microparticles were extensively characterized through scanning electron microscopy (SEM), X-ray diffraction (XRD), Fourier transform infrared spectroscopy (FTIR), differential scanning calorimetry (DSC), and thermogravimetric analysis (TGA). Drug release studies revealed a dual-stimuli response, where the release of RhB was significantly influenced by both temperature and pH. Under mildly acidic conditions (pH 4.0) at 40 °C, a rapid and complete release of RhB was observed within 120 h, while at 37 °C, the release rate was notably slower. Specifically, the release at 40 °C was 79% higher than at 37 °C, confirming the temperature sensitivity of the system. Moreover, at physiological pH (7.4), minimal drug release occurred, demonstrating the system’s potential for minimizing premature drug release under neutral conditions. This dual-stimuli approach holds promise for improving therapeutic outcomes in cancer treatment by enabling precise control over drug release in response to both pH and localized hyperthermia, reducing off-target effects and improving patient compliance.

## 1. Introduction

Metal-organic frameworks (MOFs) are a class of highly porous crystalline materials constructed from metal ions and organic ligands [1,2,3]. These materials are known for their exceptionally large surface areas, tunable pore sizes, and structural versatility, making them attractive candidates for a wide range of applications, including gas storage, catalysis, and drug delivery [4,5]. Zeolitic imidazolate framework-8 (ZIF-8), a subset of MOFs, is composed of zinc ions and methylimidazole ligands, which form a robust, highly porous structure with either rhombic dodecahedral or cubic morphologies [6,7,8]. ZIF-8’s key features—such as high porosity, low toxicity, chemical stability, and cost-effectiveness—position it as an innovative material for drug delivery systems [9,10,11]. In particular, ZIF-8’s biocompatibility and pH-sensitive degradation are critical advantages for controlled drug release [12,13]. Under acidic conditions, ZIF-8 decomposes due to the protonation of its imidazole linkers, which makes it especially suitable for targeting cancer cells and tissues that exhibit a lower pH than healthy cells [14,15,16]. This pH-triggered degradation allows for the efficient release of encapsulated therapeutic agents specifically in the tumor microenvironment [17,18,19,20,21]. Additionally, ZIF-8’s straightforward one-pot synthesis enables the encapsulation of a variety of drugs, including anticancer agents, facilitating tailored drug release profiles [22,23,24,25]. Despite its advantages, the high porosity of ZIF-8 poses challenges, as drug leakage can occur under neutral or alkaline conditions, potentially leading to premature release and unwanted side effects [26,27,28]. Efforts to mitigate this have included strategies such as surface modifications or the incorporation of additional gating materials to enhance pH selectivity and control drug release [29,30].

To address the challenge of premature drug release from ZIF-8, researchers have explored various strategies to enhance control over the release profile [31,32,33]. One approach involves encapsulating drug-loaded ZIF-8 within stimuli-responsive carriers to achieve controlled drug release triggered by specific environmental cues, such as changes in temperature or pH [34,35,36,37]. For instance, researchers have explored fatty acid-based phase change materials (PCMs) to regulate temperature- and pH-responsive drug release [38,39,40]. Ning et al. addressed the challenges of controlling the spatial and temporal release of antibacterial agents by developing a dual-stimuli responsive nanocarrier system [41]. They co-encapsulated curcumin (Cur) and indocyanine green (ICG) into ZIF-8 and polylactic acid (PLA) electrospun fibrous membranes, which were further coated with a PCM. This composite (Cur-ICG@ZIF-8/PLA/PCM) demonstrated enhanced photothermal and photodynamic stability, overcoming the typical instability of free ICG. The system enables the controlled release of curcumin via near-infrared (NIR) light-triggered PCM phase changes and ZIF-8 degradation in the acidic microenvironment of bacterial infections. In vitro and in vivo tests showed over 99% bactericidal efficacy and accelerated wound healing, providing a versatile and highly efficient platform for treating drug-resistant bacterial infections. In addition to fatty acid-based PCMs, some researchers have explored stimuli-responsive polymer coatings as alternatives to further fine-tune the responsiveness to environmental triggers [42,43,44]. Rostami et al. developed dual pH- and temperature-sensitive nanoparticles using ZIF-8 coated with chitosan-poly(*N*-isopropyl acrylamide) for co-delivery of doxorubicin (DOX) and carboplatin (CBP) in breast cancer [45]. The nanoparticles (~200 nm, +30 mV zeta potential) showed a sustained, responsive drug release. In vitro studies demonstrated significant synergy between DOX and CBP, with an IC50 of 1.96 μg/mL in MCF-7 cells and 4.54 μg/mL in MDA-MB-231 cells, while being safer for normal cells than the free drugs. This approach enhances combination therapy efficacy with reduced side effects.

Our group developed a temperature-responsive drug delivery system using ethyl cellulose (EC) nanofibers, encapsulating a eutectic mixture of lauric acid–stearic acid (LASA) as PCMs [46]. Through electrospinning, the nanofibers exhibited controlled drug release triggered by temperature changes. The system demonstrated a significant increase in drug release at 37 °C and 40 °C, highlighting its potential for smart drug delivery applications. In this study, we aimed to develop a dual-stimuli drug delivery system by directly coating a thin layer of LASA onto the surface of drug-loaded ZIF-8 microparticles. To the best of our knowledge, this is the first study of directly encapsulating drug-loaded ZIF-8 in PCM without the use of polymer matrices. This dual-responsive system ensures that drug release occurs only under targeted conditions—high temperature and low pH—thereby preventing premature release and minimizing potential side effects. To synthesize the core-shell microparticles, we utilized coaxial electrospraying (Co-ES), an advanced electrohydrodynamic atomization technique that enables the formation of well-defined core-shell structures [47]. In our design, ZIF-8 loaded with rhodamine B (RhB), a fluorescent dye commonly used as a model drug and tracer [48,49,50], forms the core [51]. RhB is encapsulated within ZIF-8 through a one-pot synthesis method, ensuring uniform drug distribution within the MOF. The shell is composed of LASA, which forms a protective layer around the drug-loaded ZIF-8, creating a thermally and pH-responsive microparticle.

## 2. Result and Discussion

### 2.1. Synthesis of ZIF-8 and RhB@ZIF-8

**Figure 1:**
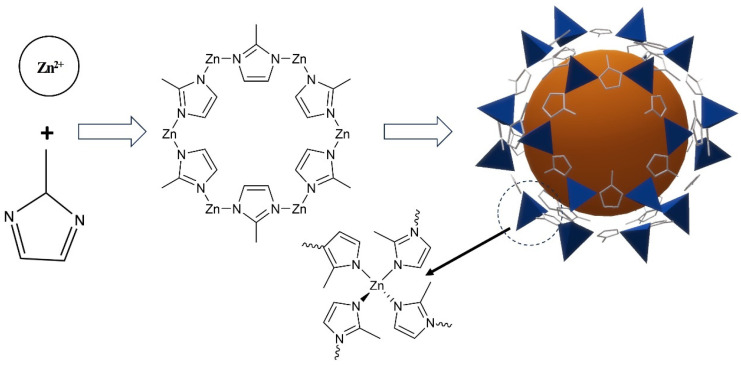
Schematic representation of the ZIF-8 synthesis process.

**Figure 2:**
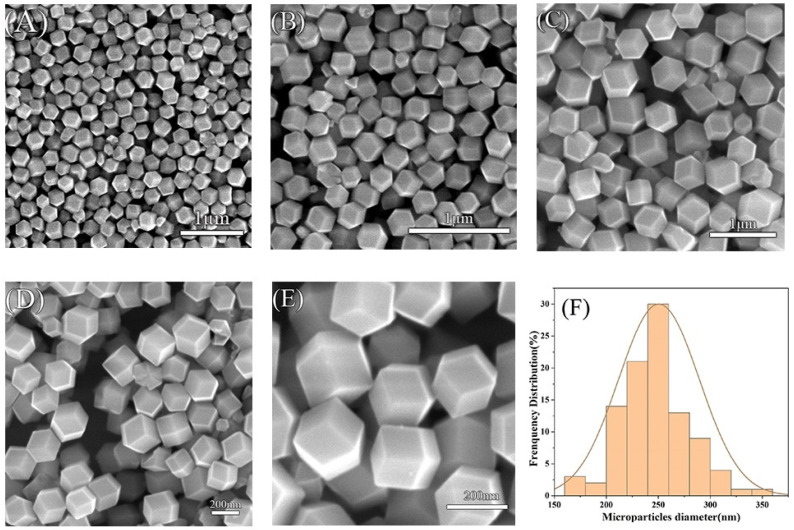
SEM images of ZIF-8 at various magnifications. (**A**) 10,000×, (**B**) 20,000×, (**C**) 30,000×, (**D**) 50,000×, and (**E**) 100,000×. These images highlight the rhombic dodecahedral and cubic morphologies of the particles. (**F**) Diameter distribution of ZIF-8 particles.

ZIF-8 was synthesized by mixing zinc acetate dihydrate and 2-methylimidazole in a molar ratio of 35:1 in purified water. The synthetic scheme is illustrated in Figure 1. In this process, zinc ions and organic ligands were prepared separately in deionized water, followed by their combination to form ZIF-8 [52]. ZIF-8 typically forms in either cubic or rhombic dodecahedral shapes, as shown in Figure 2, which presents SEM images at magnifications ranging from 10 K to 100 K. In Figure 2A, the majority of ZIF-8 crystals exhibit a rhombic dodecahedral shape, while a minority display a cubic morphology. Some smaller microparticles appear less well-defined, likely due to incomplete formation of the rhombic dodecahedral shape during synthesis (Figure 2B–E). This may be attributed to the solvent selection, as DI water was used in this study to maintain consistency with RhB@ZIF-8 synthesis. However, it is noted that methanol is a more suitable solvent for the synthesis of well-formed ZIF-8 crystals [53]. Additionally, sonication during the washing step might have caused partial fragmentation of the particles [54]. The average diameter of ZIF-8 particles was measured to be 251 nm, with a size range spanning from 25 nm to 296 nm. The size distribution, as depicted in Figure 2F, is based on measurements of more than 100 representative microparticles, ensuring statistical accuracy. The results reveal that 64% of ZIF-8 particles have diameters concentrated between 220 nm and 278 nm, confirming a high uniform size distribution.

**Figure 3:**
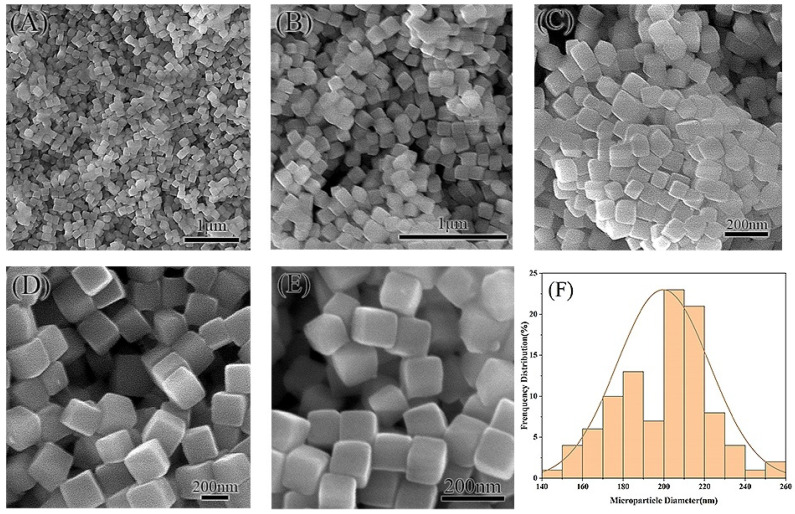
SEM micrographs and particle size distribution of RhB@ZIF-8 microparticles. Images (**A**) through (**E**) display the morphology of RhB@ZIF-8 at varying magnifications: (**A**) 10,000×, (**B**) 20,000×, (**C**) 30,000×, (**D**) 50,000×, and (**E**) 100,000×. The micrographs reveal uniform rhombic dodecahedron-shaped microparticles with smooth surfaces, typical of ZIF-8 structures. Plot (**F**) shows the corresponding particle diameter distribution of RhB@ZIF-8, demonstrating a relatively narrow size distribution with an average diameter centered around 200 nm.

RhB@ZIF-8 was synthesized by incorporating a concentrated RhB solution during the formation of ZIF-8. The RhB stock solution was prepared in purified water to maintain consistency with the solvent used in ZIF-8 synthesis. The one-pot synthesis method employed for encapsulating RhB within ZIF-8 is both efficient and scalable, allowing for large-scale production of RhB@ZIF-8 particles. SEM images of RhB@ZIF-8 at varying magnifications (10 K, 20 K, 30 K, 50 K, and 100 K) are shown in Figure 3. Notably, all RhB@ZIF-8 structures display a cubic morphology, distinct from the rhombic dodecahedral shape observed in pure ZIF-8 (Figure 2). The diameter distribution analysis (Figure 3F) indicates that 64% of RhB@ZIF-8 particles have diameters ranging from 180 nm to 220 nm, with an average diameter of 200 nm, based on measurements of more than 100 representative particles. Compared to ZIF-8, RhB@ZIF-8 exhibits a smaller particle size. The incorporation of RhB appears to reduce the diameter and influence the morphology, likely inhibiting crystal growth and leading to a more cubic structure. The cubic shape may represent an intermediate stage in the formation of the rhombic dodecahedral structure typical of ZIF-8 [55]. The presence of RhB during synthesis likely affects the nucleation and growth kinetics of ZIF-8, leading to these observed morphological differences.

To quantify the RhB content, a calibration curve was constructed by measuring the absorbance of RhB at concentrations ranging from 0 to 10 μg/mL (The calibration equation: Y = 0.206X + 0.0189, with an R^2^ value of 0.992). The high R^2^ value confirms the reliability of the calibration curve for accurately determining the RhB concentration in measurements (Figure S1). Although the particles were thoroughly washed, trace amounts of RhB may still remain on their surface. However, this residual RhB is present in negligible quantities and is unlikely to significantly affect the calculation of encapsulation efficiency. The encapsulation efficiency, determined by the mass of RhB encapsulated within a given quantity of RhB@ZIF-8 (Equation (1)), was 6.28% (6.28 mg of RhB per 100 mg of particles).

### 2.2. Structure of RhB@ZIF-8@PCM 

To develop a pH- and temperature-responsive drug delivery system, it is critical to incorporate temperature-responsive materials like LASA (lauric acid-stearic acid, a mixture of long-chain fatty acids) into the design. The deposition of LASA onto RhB@ZIF-8 microparticles was achieved through coaxial electrospraying, a method that allows for precise core-shell structuring. In this process, a small amount of poly (lactic acid) (PLA) was utilized due to its dual role in binding LASA to the microparticles and its biodegradable nature. PLA is an ideal binder because it facilitates the formation of a uniform coating on the RhB@ZIF-8 microparticles, while also offering biodegradable properties that enable it to degrade into non-toxic metabolites, such as lactic acid, in the human body. Moreover, PLA enhances the drug carrier’s characteristics, contributing to controlled drug release profiles. LASA was used at a concentration of 0.2 g/mL, with chloroform serving as the non-polar solvent of choice. Chloroform’s high efficacy in dissolving fatty acids and its ability to form homogeneous solutions made it suitable for this application. Additionally, its low boiling point, chemical stability, and high volatility supported rapid evaporation during the coaxial electrospraying process, ensuring the formation of a stable LASA coating on the microparticles.

**Figure 4:**
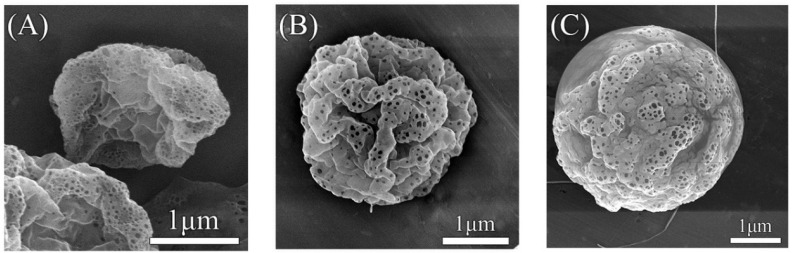
SEM images of PLA microparticles at various concentrations of PLA in dichloromethane (DCM) under 20,000× magnification: (**A**) 1 wt% PLA in DCM, showing shriveled particles with prominent surface pores; (**B**) 2 wt% PLA in DCM, displaying fuller particles with fewer wrinkles and visible pores; (**C**) 3 wt% PLA in DCM, showing nearly spherical particles with minimal wrinkles and fewer pores compared to lower concentrations. The increase in PLA concentration results in more uniform and spherical microparticle morphology.

Figure 4 illustrates the morphological changes of PLA microparticles as the concentration of PLA in the DCM solution increases. In Figure 4A, corresponding to the lowest concentration (1 wt% PLA), the microparticles exhibit a shriveled appearance with significant surface pores, indicative of particle shrinkage during the drying process. This suggests incomplete formation or poor particle integrity at lower concentrations. In Figure 4B, with a concentration twice that of Figure 4A (2 wt% PLA), the microparticles appear fuller and more structured. However, the presence of surface wrinkles and pores persists, though less pronounced than at lower concentrations. This indicates an improvement in particle morphology with increasing PLA concentration but still reveals some imperfections. In Figure 4C, representing the highest concentration (3 wt% PLA), the microparticles show the most spherical and uniform structure. Although some surface pores are still visible, the overall particle integrity is significantly enhanced compared to Figure 4A,B. The particles exhibit minimal wrinkles, indicating that the higher concentration of PLA promotes better formation of the microparticles. Thus, under a 25 kV electric field, the highest concentration of PLA (3 wt%) produces the best-quality microparticles, with a more consistent and spherical morphology. However, for the encapsulation of RhB@ZIF-8, the PLA concentration needs to be carefully optimized further. If the concentration is too high, the formation of fibers or polymer beads could occur instead of uniform microparticles. Based on comparative experiments with coaxial electrospraying, a concentration around 1 wt% PLA is expected to be optimal for encapsulating RhB@ZIF-8, balancing particle formation and encapsulation efficiency.

**Figure 5:**
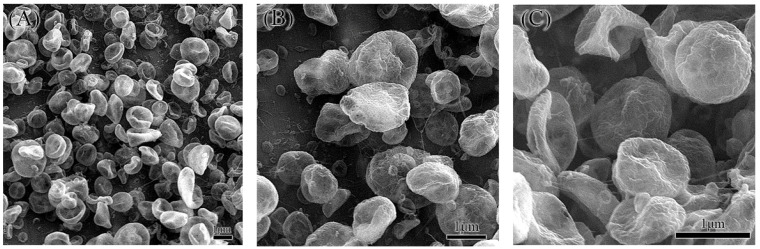
SEM images of RhB@PCM microparticles at varying magnifications in DCM: (**A**) 5000× magnification, showing the overall surface morphology with irregular and wrinkled structures; (**B**) 10,000× magnification, providing a closer view of the individual microparticles, revealing shrinkage and deformation of the particle surfaces; and (**C**) 20,000× magnification, displaying detailed surface texture and porosity, highlighting the structural inconsistencies and the non-uniformity of the particle surfaces.

Controlled experiments allow for the manipulation of various influencing factors, enabling precise determination of causal relationships while minimizing biases. In this study, coaxial electrospraying was used to synthesize microparticles with a shell of LASA and a core of RhB. Unlike the dual-responsive RhB@ZIF-8@PCM, the RhB@PCM microparticles lack ZIF-8 and display only temperature-responsive behavior. For these experiments, the inner solution consisted of a 1 wt% PLA solution containing 100 µg of RhB, rather than a suspension of RhB@ZIF-8. The SEM images in Figure 5 show the morphology of RhB@PCM microparticles at 5000×, 10,000×, and 20,000× magnifications. Compared to PLA-only microparticles, RhB@PCM exhibits a non-porous surface due to the presence of the LASA shell, which effectively fills the PLA pores. In the absence of ZIF-8, the microparticles appear shriveled and less voluminous, with smoother and more uniform surfaces. This structural shrinkage is likely caused by the lack of the rigid ZIF-8 framework, which usually provides greater structural integrity.

**Figure 6:**
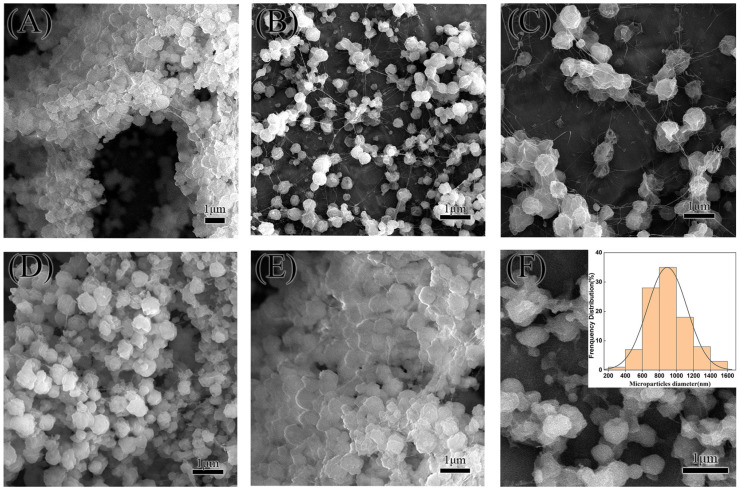
SEM images of RhB@ZIF-8@PCM microparticles at varying magnifications: (**A**,**B**) show the surface morphology of RhB@ZIF-8@PCM microparticles at 5000× magnification, highlighting their clustered structure and partial aggregation; (**C**–**F**) show the same particles at 10,000× magnification, revealing more detailed structural features, including the presence of fine fibers and surface irregularities. (**F**) includes the diameter distribution plot, indicating a relatively narrow size range with a peak centered around 892 nm.

Figure 6 presents SEM images of RhB@ZIF-8@PCM microparticles at magnifications of 5000× and 10,000×. The images reveal that even with the PLA concentration reduced to 1 wt%, fiber formation still occurs, as evidenced by the fine fibers visible in panels (A) and (F). Despite the presence of fibers, a substantial number of spherical microparticles were successfully produced, with an average diameter of approximately 892 nm. This is significantly larger than the diameter of RhB@ZIF-8 particles alone, which suggests that each RhB@ZIF-8@PCM microparticle encapsulates multiple RhB@ZIF-8 particles within its structure. The particle diameter distribution, shown in Figure 6F, indicates that 63% of the microparticles have diameters between 600 nm and 1000 nm. This distribution was calculated based on measurements of over 100 particles, ensuring statistical accuracy. The SEM images further reveal that the particles tend to aggregate upon drying, with a continuous layer of solid LASA coating visible on their surfaces. This aggregation may be attributed to the nature of the drying process, as well as the cohesive properties of the LASA shell material.

**Figure 7:**
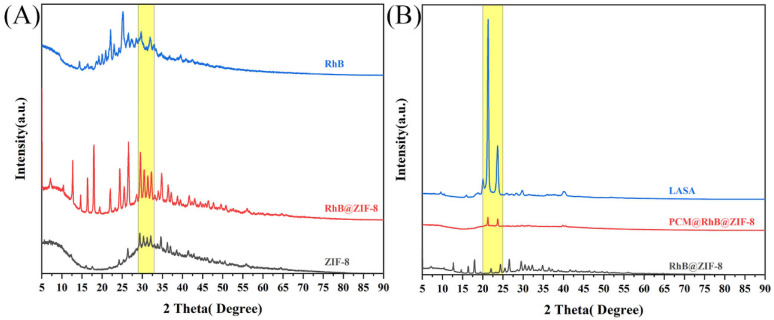
XRD patterns illustrating the crystallographic structures of the various components. (**A**) shows the diffraction patterns of RhB, ZIF-8, and RhB@ZIF-8, highlighting the characteristic peaks of each material. (**B**) presents the XRD patterns of LASA, RhB@ZIF-8, and RhB@ZIF-8@PCM.

Figure 7A presents the XRD analysis of RhB, ZIF-8, and RhB@ZIF-8, revealing the crystalline characteristics of these materials. Each sample exhibits multiple diffraction peaks, indicating their crystalline nature. The highest peaks for ZIF-8 are observed at 29.3°, 30.2°, 31.2°, and 32°, characteristic of its well-defined crystal structure [56]. In RhB@ZIF-8, these peaks remain prominent, suggesting that the encapsulation of RhB does not disrupt the primary crystalline structure of ZIF-8. For RhB, the strongest peaks are observed at 22.1° and 25.1°, confirming its crystalline form [46]. In the RhB@ZIF-8 composite, similar peaks at 22.1° and 26° confirm the successful encapsulation of RhB within ZIF-8. Additionally, noticeable peaks at 17.9°, 12.7°, and 16.4° in RhB@ZIF-8 indicate structural changes due to the incorporation of RhB. These shifts in peak positions and intensities between ZIF-8 and RhB@ZIF-8 reflect the interactions between the RhB molecules and the ZIF-8 framework, which may influence the overall crystal structure. In Figure 7B, the XRD pattern of LASA shows prominent peaks at 21.3° and a less intense peak at 23.6°, characteristic of its crystalline phase [57]. The XRD pattern for RhB@ZIF-8@PCM does not show significant peaks corresponding to RhB@ZIF-8 or ZIF-8, indicating that RhB@ZIF-8 is effectively encapsulated within the PCM [58]. The peaks at 21.2° and 23.7° are consistent with the LASA shell coating, further confirming the successful encapsulation of RhB@ZIF-8 within the LASA matrix.

**Figure 8:**
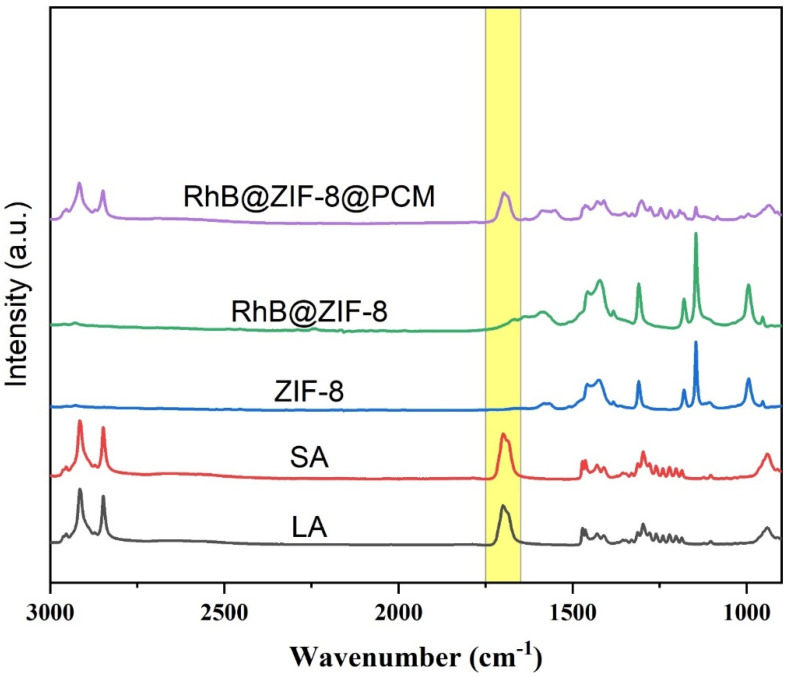
FTIR spectra of LA, SA, ZIF-8, RhB@ZIF-8, and RhB@ZIF-8@PCM.

FTIR is a widely used spectroscopic technique for chemical analysis, providing insights into molecular structures, including chemical bonds and functional groups [38,59]. Figure 8 presents the FTIR spectra of LA, SA, ZIF-8, RhB@ZIF-8, and RhB@ZIF-8@PCM, highlighting key functional groups in each component. In the spectra of LA and SA, strong peaks appear between 1666 cm^−1^ and 1737 cm^−1^, with the peak at 1696 cm^−1^ being particularly notable, indicating the presence of carbonyl (C=O) bonds typical of carboxylic acid in both fatty acids [60]. These carbonyl peaks confirm the existence of ester functional groups, important for the role of PCM. Additionally, the presence of C–H stretching vibrations in both LA and SA is indicated by strong peaks in the region between 2990 cm^−1^ and 2850 cm^−1^ [61]. Specifically, Figure 8 shows prominent peaks at 2913 cm^−1^ and 2847 cm^−1^, corresponding to the symmetric and asymmetric stretching of methylene (C–H) groups, which are characteristic of long-chain fatty acids [62]. For ZIF-8, characteristic peaks appear in the range of 1500–1000 cm^−1^, representing the C=N and C–N stretching vibrations from the imidazole rings in the ZIF-8 framework [63]. These peaks are also observed in the spectra of RhB@ZIF-8 and RhB@ZIF-8@PCM, confirming the integrity of the ZIF-8 structure even after RhB encapsulation and coating with PCM. In RhB@ZIF-8@PCM, the FTIR spectra show the expected peaks for LA and SA, including the C=O and C–H bonds, confirming the successful inclusion of the PCM layer. Additionally, two prominent peaks at 1145 cm^−1^ and 995 cm^−1^ correspond to C=N and C–N stretching vibrations in the imidazole ring, indicating the presence of ZIF-8 within the composite [64]. The consistent presence of these functional groups confirms the successful encapsulation of RhB@ZIF-8 within the PCM matrix, with no significant alterations to the molecular structures [65].

### 2.3. Model Drug Release Kinetics 

**Figure 9:**
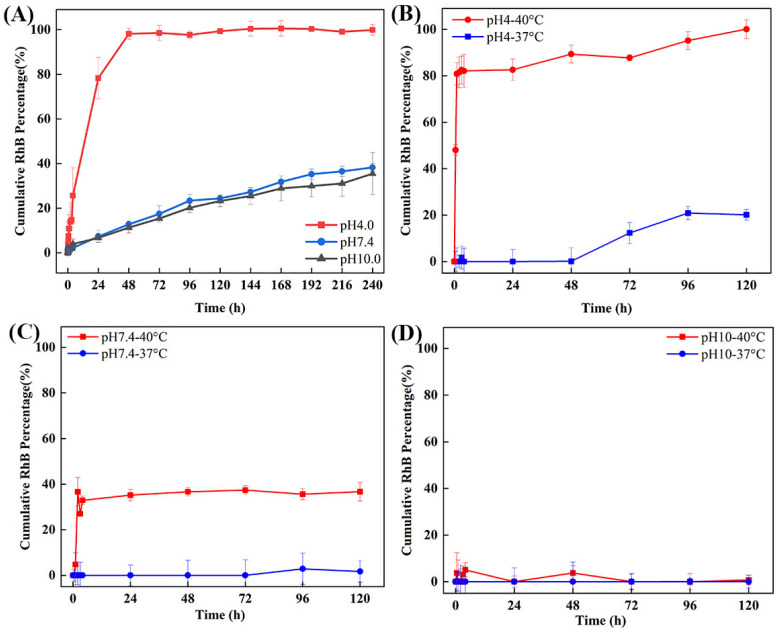
Cumulative RhB release profiles under different pH and temperature conditions. (**A**) RhB release from RhB@ZIF-8 at pH 4.0, 7.4, and 10.0 at 37 °C, showing rapid release at pH 4.0 and slower, sustained release at neutral and basic pH values. (**B**) RhB release from RhB@ZIF-8@PCM at pH 4.0 under 37 °C and 40 °C, demonstrating a significantly higher release rate at the elevated temperature. (**C**) RhB release from RhB@ZIF-8@PCM at pH 7.4 under 37 °C and 40 °C, with a noticeable increase in release at 40 °C compared to 37 °C. (**D**) RhB release from RhB@ZIF-8@PCM at pH 10.0 under 37 °C and 40 °C, showing minimal release at both temperatures, indicating stability under basic conditions.

To investigate the chemical properties of ZIF-8 under acidic conditions, Figure 9A shows the in vitro release profile of RhB@ZIF-8 at different pH levels at 37 °C. At pH 4.0, RhB@ZIF-8 released up to 92.58% of its RhB content within the first 48 h, reaching 99.30% by 120 h. This rapid release is due to the acidic environment breaking down the ZIF-8 framework, allowing the complete release of encapsulated RhB. Under neutral (pH 7.4) and basic (pH 10.0) conditions, the release was significantly slower, with 12.79% and 11.33% released at 48 h, eventually reaching 38.29% and 35.5% by 240 h, respectively. These differences suggest that ZIF-8 remains stable at neutral and basic pH, with drug release occurring primarily through the gradual diffusion of RhB through the ZIF-8 pores. The release profile showed minimal standard deviation across three experiments, confirming the reproducibility of the results. The rapid degradation of ZIF-8 under acidic conditions leads to near-complete drug release, whereas at neutral and basic pH, RhB is released slowly, likely due to pore diffusion. This highlights the pH-responsive behavior of ZIF-8. The room temperature data, which served as a baseline for these experiments, recorded an average release of ~26.24% ± 3.1% at pH 7.4 over 120 h. This baseline suggests partial RhB penetration from the ZIF-8 structure due to incomplete encapsulation or potential damage to the particle shells during processing. These factors could explain the slow release of RhB under neutral conditions.

Figure 9B shows the release profile of RhB@ZIF-8@PCM at 37 °C and 40 °C under acidic conditions (pH 4.0). At 40 °C, the release was rapid, reaching 82.08% in the first 4 h and 100% by 120 h. In contrast, at 37 °C, only 12.32% was released by 72 h, with a gradual increase to 20.08% by 120 h. The increased release rate at 40 °C suggests that the phase change material (PCM) undergoes a transition, facilitating faster drug release. DSC and TGA were conducted to evaluate the thermal properties and stability of the synthesized materials (Figure S2). The DSC analysis of PCM@RhB@ZIF-8 revealed a melting point of 38.6 °C for the LASA eutectic mixture (Figure S2A), slightly lower than the melting point of 39.1 °C observed for pristine LASA (Figure S2B). This reduction in melting point may be attributed to the interactions between LASA and the ZIF-8 framework, which can alter the thermal behavior of the encapsulated material. TGA analysis further assessed the thermal stability of ZIF-8, RhB@ZIF-8, LASA, and PCM@RhB@ZIF-8. The results demonstrated that ZIF-8 and LASA are thermally stable under the conditions relevant to their intended applications (Figure S2C). Figure 9C illustrates the release profile of RhB@ZIF-8@PCM at pH 7.4, showing a temperature-dependent release. At 40 °C, the release reached 39% within the first 3 h, followed by a slight fluctuation and stabilization. This fluctuation may be attributed to instrumental or sampling errors, or to evaporation during the release experiment. Repeated experiments at 40 °C showed a release plateau at ~36.6% by 120 h, while the release at 37 °C remained minimal (around 1%). These results suggest that the LASA shell may not fully enclose the RhB@ZIF-8 core, resulting in some RhB leakage. Figure 9D shows the release profile under basic conditions (pH 10.0) at both 37 °C and 40 °C. At 40 °C, 6.96% of RhB was released by 120 h, whereas at 37 °C, only 0.6% was released in the same period. These minimal release rates indicate that the structure of RhB@ZIF-8@PCM is stable under basic conditions, with limited degradation or diffusion. Minor releases observed at the fourth and forty-eighth hours at 37 °C may be due to experimental errors, including inconsistencies in instrument measurements or sample handling. The results from these experiments suggest that RhB@ZIF-8@PCM exhibits pH- and temperature-responsive release behavior. The structure is stable under neutral and basic conditions, with slow diffusion-driven release. However, under acidic conditions, especially at higher temperatures, the PCM shell facilitates rapid drug release. The differences in release profiles across different conditions reflect the importance of both the ZIF-8 framework and the PCM layer in controlling drug delivery.

**Figure 10:**
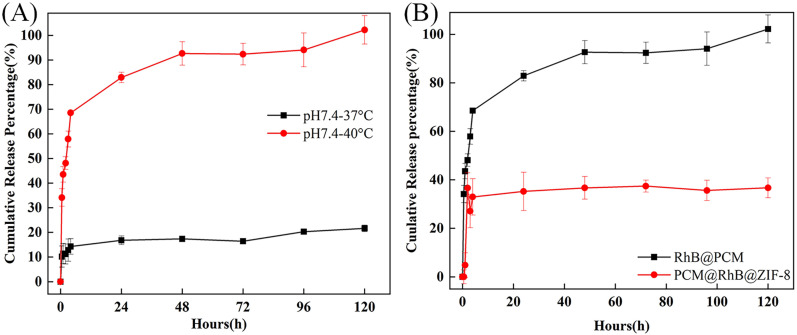
Cumulative RhB release profiles under different conditions. (**A**) Release of RhB from RhB@PCM at pH 7.4 and temperatures of 37 °C and 40 °C, showing a significantly higher release rate at 40 °C compared to 37 °C. (**B**) Comparison of RhB release from RhB@PCM and RhB@ZIF-8@PCM at 40 °C and pH 7.4, highlighting the faster release from RhB@PCM due to the absence of the ZIF-8 core, while RhB@ZIF-8@PCM shows a more controlled and sustained release due to the ZIF-8 framework.

Figure 10A illustrates the release profile of RhB@PCM in pH 7.4 at 37 °C and 40 °C, confirming the temperature-responsive behavior of the LASA shell. At 40 °C, the release rapidly increases within the first four hours, reaching 68%, and then gradually progresses to 100% over 120 h. The rapid release during the initial phase followed by a slower release rate suggests that the phase change of LASA at 40 °C facilitates quick RhB diffusion, followed by a sustained release over time. Conversely, at 37 °C, the release is much slower, reaching only 14.23% in the first four hours and increasing to 21.6% by 120 h. The lower release at 37 °C, which is 79% less than at 40 °C over the same time frame, highlights the role of LASA as a temperature-responsive material. The higher temperature likely causes the LASA shell to undergo a phase transition, allowing for a more efficient and complete release of RhB. These findings demonstrate that the coaxial electrospraying technique effectively encapsulates LASA as a shell around the RhB core. As temperature increases, LASA’s phase change facilitates rapid and complete RhB release, making RhB@PCM a highly temperature-responsive microparticle system. This temperature sensitivity could be advantageous in applications where controlled release is needed at elevated temperatures, such as targeted drug delivery in hyperthermic conditions.

Figure 10B compares the cumulative RhB release profiles from RhB@PCM and RhB@ZIF-8@PCM at 40 °C under neutral pH (pH 7.4). The data clearly demonstrate distinct release behaviors between the two systems. In the case of RhB@PCM, the release profile shows a rapid increase in the first few hours, reaching approximately 85% cumulative release by 24 h, and gradually approaching 100% by 120 h. This rapid release is attributed to the absence of the ZIF-8 framework, allowing the encapsulated RhB to diffuse out of the PCM layer at an accelerated rate. The LASA shell in RhB@PCM is highly responsive to temperature changes, and at 40 °C, the shell undergoes a phase transition, promoting quick drug release. The absence of the rigid ZIF-8 core leads to the faster diffusion of RhB molecules. In contrast, RhB@ZIF-8@PCM exhibits a much slower and more controlled release. The cumulative release reaches only about 38% by 120 h. The ZIF-8 core in this composite serves as a framework that slows down the release of RhB, even at an elevated temperature of 40 °C. The ZIF-8 structure provides additional resistance to the diffusion of RhB, resulting in a more sustained release profile. The presence of ZIF-8 prevents the immediate release seen with RhB@PCM, as RhB must first diffuse through the porous ZIF-8 structure before exiting the PCM shell. This comparison highlights the critical role that the ZIF-8 core plays in modulating the release kinetics of RhB. While RhB@PCM releases almost entirely within 120 h due to the temperature-sensitive PCM layer, RhB@ZIF-8@PCM shows a more controlled, extended release profile due to the encapsulation within the ZIF-8 framework. The combination of the PCM and ZIF-8 in RhB@ZIF-8@PCM offers a dual mechanism for controlled release, making it a promising system for applications requiring sustained drug delivery over extended periods.

## 3. Experiment Section

### 3.1. Chemical and Material 

Zinc acetate dihydrate (Zn(O_2_CCH_3_)_2_·2H_2_O, 98.0–101.0%, BeanTown Chemical, Hudson, NH, USA), a metal salt comprising zinc, acetic acid, and two water molecules, was used as the metal precursor. 2-Methylimidazole (2-Hmim, 99%) from Sigma-Aldrich, St. Louis, MO, USA, an organic imidazole compound, served as the ligand in the synthesis of ZIF-8. Rhodamine B (RhB, ≥95%, verified by HPLC), a model drug dye, and two naturally occurring fatty acids, lauric acid (LA, ≥98%) and stearic acid (SA, 97%), also sourced from Sigma-Aldrich. LA and SA, in a 4:1 ratio, were used to prepare the temperature-responsive phase change material (PCM). Ethanol (200 proof, ACS grade, VWR) and anhydrous *N*,*N*-Dimethylformamide (≥99.9%, VWR) were used as solvents for dissolving LA and SA. Polylactic acid (PLA, GoodFellow, Pittsburgh, PA, USA) was incorporated in a low concentration in fatty acid solution to enhance the electrospray process and improve the stability of the core-shell microparticles. Purified water was generated from a Millipore Direct-Q 8 UV water purification system (resistivity of 8.2 MΩ·cm at 25 °C).

### 3.2. Synthesis of ZIF-8

A stock solution of zinc acetate dihydrate (Zn(O_2_CCH_3_)_2_·2H_2_O) was prepared by dissolving 0.293 g of the salt in 10 mL of purified water, yielding a concentration of 29.3 mg/mL. Separately, 1.08 g of 2-methylimidazole (2-mim) was dissolved in 20 mL of purified water to achieve a concentration of 54 mg/mL. The 2-mim solution was then added dropwise to the zinc acetate solution while stirring continuously on a magnetic stir plate. The reaction mixture was stirred overnight to ensure complete synthesis of ZIF-8. The resulting precipitate was collected by centrifugation at 10,000 rpm for 10 min. The precipitate was thoroughly washed with deionized water and ethanol to remove any residual reactants. The purified ZIF-8 powder was then dried under vacuum overnight to remove any remaining moisture, yielding the final product.

### 3.3. Synthesis of Rhodamine B-Encapsulated ZIF-8 (RhB@ZIF-8)

**Figure 11:**
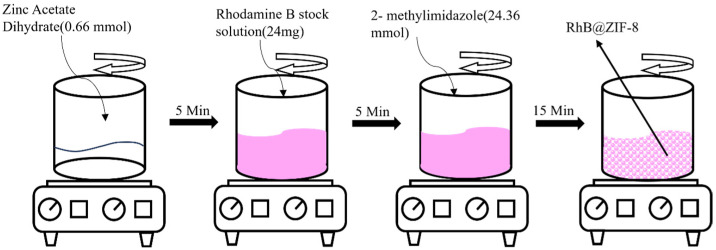
Schematic representation of the stepwise synthesis process for RhB@ZIF-8.

A stock solution of rhodamine B (RhB) was prepared at a concentration of 6 mg/mL in purified water. Separately, 0.200 g of zinc acetate dihydrate was dissolved in 0.8 mL of purified water (0.66 mmol). To this solution, 4 mL of the RhB solution was added, and the mixture was stirred for 5 min at room temperature. A solution of 2.000 g of 2-methylimidazole (2-mim) was then prepared by dissolving it in 4 mL of purified water (24.36 mmol). The 2-mim solution was added dropwise to the zinc acetate and RhB mixture while continuous stirring was maintained. After 15 min, the precipitate was collected by centrifugation at 10,000 rpm for 10 min. The precipitate was washed three times with a mixture of ethanol and water to remove any residual reactants. The washed product was then dried under vacuum at room temperature to obtain the pink RhB@ZIF-8 powder, as shown in Figure 11.

To determine the encapsulation efficiency, approximately 20 mg of RhB@ZIF-8 was weighed and dissolved in 20 μL of 2 M HCl. The solution was then diluted with pH 7.4 phosphate-buffered saline (PBS), and the absorbance was measured at 544 nm using UV-Vis spectrophotometry. A calibration curve was used to quantify the amount of RhB in the sample. The encapsulation efficiency was calculated using Equation (1):

(1) $$Encapsulate\;Efficiency=\frac{Mass\;of\;RhB\;in\;\text{ZIF-8}}{Total\;Mass\;of\;RhB@\text{ZIF-8}}\times 100\%$$

### 3.4. Drug Release from RhB@ZIF-8

Phosphate-buffered saline (PBS) is commonly used to assess the efficacy and safety of nanoparticulate drug delivery systems. In this study, drug release experiments were conducted using RhB@ZIF-8 in a PBS-based system. Approximately 100 ± 5 mg of RhB@ZIF-8 was suspended in 20 mL of pre-warmed PBS at varying pH levels (pH 4, pH 7.4, and pH 10). Each suspension was maintained at 37 °C in a 50 mL centrifuge tube, which was pre-heated using a water bath. At predetermined time intervals, 1 mL aliquots were collected from the suspension and immediately diluted to 3 mL with PBS buffer. UV-Vis spectroscopy was used to measure the amount of RhB released into the solution by monitoring absorbance at the corresponding wavelength. The release percentage of rhodamine B was calculated using Equation (2). Each release experiment was repeated at least three times to obtain average values. The release profile was plotted based on the average release percentages at each time point.

(2) $$Release\;Percentage=\frac{Amount\;of\;RhB\;released}{Total\;amount\;of\;RhB\;loaded}\times 100\%$$

### 3.5. Encapsulation of RhB@ZIF-8 in LASA Microparticles Using Coaxial Electrospraying

**Figure 12:**
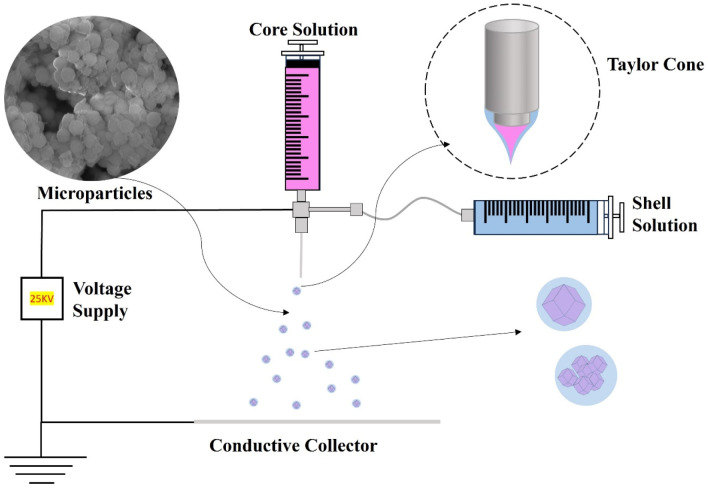
Schematic illustration of the coaxial electrospraying experimental setup for the production of core-shell microparticles (RhB@ZIF-8@PCM).

The coaxial electrospraying setup consisted of two syringes, two programmable syringe pumps (Legato 110, KD Scientific, Holliston, MA, USA), tubing, a high-voltage power supply (ES30P-5W, Gamma High Voltage Research, Ormond Beach, FL, USA), a flat conductive collector (aluminum foil), and a coaxial blunt metal needle (Figure 12). To improve stability, a small amount of PLA (~1%) was added to the core solution containing RhB@ZIF-8. This core solution was loaded into the syringe connected to the inner needle of the coaxial spinneret, with a flow rate of 1 mL/h. The shell solution consisted of PCM, prepared by dissolving 1.6 g of LA and 0.4 g of SA in 10 mL of chloroform (0.2 g/mL) at a 4:1 mass ratio. This solution was loaded into a syringe for the outer shell and dispensed at a flow rate of 1 mL/h. The coaxial electrospraying was conducted with a tip-to-collector distance of 15 cm and an applied voltage of 25 kV. The relative humidity was maintained at 40 ± 2%. This setup and operation parameters provided stable electric field conditions, optimizing the formation of RhB@ZIF-8@PCM core-shell microparticles through efficient spraying and encapsulation. For comparison, RhB@PCM microparticles were also synthesized using the same coaxial electrospraying method, with pure RhB replacing RhB@ZIF-8 in the core solution.

### 3.6. In Vitro Drug Release

The in vitro drug release experiments were conducted using PBS at three different pH levels (pH 4.0, pH 7.4, and pH 10) and temperatures (37 °C, 40 °C, and ambient room temperature). Approximately 10 ± 0.4 mg of RhB@ZIF-8@PCM or RhB@PCM were dispersed in 20 mL of pre-warmed PBS in 50 mL centrifuge tubes. The samples were incubated in a water bath to maintain the specified temperatures (37 °C, 40 °C, and ambient conditions). The experimental conditions are detailed in Table 1. At predetermined time intervals, 1 mL of the PBS solution was collected from each tube and diluted with PBS to a final volume of 3 mL. The concentration of released RhB in the collected samples was measured using a UV/Vis spectrometer (PerkinElmer, Waltham, MA, USA, Lambda 35), based on a pre-established calibration curve. The release percentage of RhB at each time point was calculated by Equation (2).

### 3.7. Characterizations

Various advanced characterization techniques were employed to analyze the synthesized samples. UV/Vis spectroscopy (190–800 nm) was utilized to quantitatively measure the RhB concentration in PBS, aiding in the determination of the encapsulation efficiency and release percentage. Scanning electron microscopy (SEM, ThermoFisher Apreo FEI, Waltham, MA, USA) provided high-resolution imaging for size and morphological analysis of the ZIF-8, RhB@ZIF-8, RhB@ZIF-8@PCM, and RhB@PCM particles, with samples coated in a conductive layer to enhance image quality. Particle size distribution was measured using ImageJ software (Version 1.54k) from particles in representative SEM images. X-ray diffraction (XRD, Bruker D8 Discover, Bruker Corporation, Billerica, MA, USA) was used to determine crystallographic structures over a 2θ range of 5° to 90°, identifying crystal phases, morphology, and purity of the samples. Fourier transform infrared spectroscopy (FTIR) was employed to identify chemical bonds and functional groups, revealing key molecular interactions. Thermal properties of the materials were investigated through differential scanning calorimetry (DSC), which measured heat flow during phase transitions to determine critical thermal events like melting and recrystallization. Thermogravimetric analysis (TGA, TA SDT Q600, New Castle, DE, USA) was used to monitor mass changes as a function of temperature, providing insights into the compositional stability of the samples under a nitrogen atmosphere.

## 4. Conclusions

This study successfully demonstrated the synthesis and characterization of a dual-stimuli-responsive drug delivery system using RhB-encapsulated ZIF-8 (RhB@ZIF-8) and PCM. The core-shell microparticles, fabricated using coaxial electrospraying, incorporated RhB@ZIF-8 within a eutectic mixture of LA and SA as the temperature-responsive PCM. Detailed structural and morphological analyses confirmed the integrity of the ZIF-8 core and the uniform coating provided by the PCM shell. The in vitro drug release experiments revealed that the RhB@ZIF-8@PCM system exhibited a strong response to both pH and temperature triggers, making it highly effective for controlled drug release. Under acidic conditions (pH 4.0), the ZIF-8 framework degraded rapidly, facilitating complete drug release within 120 h. Temperature sensitivity was confirmed as the PCM shell underwent phase transitions at elevated temperatures, leading to significantly faster drug release at 40 °C compared to 37 °C. Furthermore, the RhB@ZIF-8@PCM system demonstrated excellent stability under neutral and basic conditions, with minimal drug release observed at pH 7.4 and 10.0. This dual-stimuli responsiveness ensures that the drug remains encapsulated under physiological conditions, preventing premature release and potential side effects. The RhB@ZIF-8@PCM core-shell microparticles offer a promising approach for targeted drug delivery, particularly in hyperthermic environments such as cancerous tissues. The combination of pH-sensitive ZIF-8 and temperature-responsive PCM provides precise control over drug release, enhancing therapeutic outcomes while minimizing off-target effects. Future studies will explore further optimizations in particle morphology and encapsulation efficiency to enhance the scalability and applicability of this drug delivery system in in vitro and in vivo settings.

## Figures and Tables

**Table 1:** In vitro drug release conditions for RhB@ZIF-8@PCM and RhB@PCM microparticles.

Samples	Temperature (°C)	pH
RhB@ZIF-8@PCM	37 °C	4.0
RhB@ZIF-8@PCM	40 °C	4.0
RhB@ZIF-8@PCM	Room temperature	4.0
RhB@ZIF-8@PCM	37 °C	7.4
RhB@ZIF-8@PCM	40 °C	7.4
RhB@ZIF-8@PCM	Room temperature	7.4
RhB@ZIF-8@PCM	37 °C	10.0
RhB@ZIF-8@PCM	40 °C	10.0
RhB@ZIF-8@PCM	Room temperature	10.0
RhB@PCM	37 °C	7.4
RhB@PCM	40 °C	7.4
RhB@PCM	Room temperature	7.4

## Data Availability

The data that support the findings of this study are available from the corresponding author upon reasonable request.

## Supplementary Materials

The following supporting information can be downloaded at: https://www.mdpi.com/article/10.3390/molecules29225291/s1, Figure S1: Calibration curve of RhB in phosphate-buffered saline (PBS); Figure S2: DSC and TGA thermograms of different samples.
