# Supplementary material for: Engineering pH and Temperature-Triggered Drug Release with Metal-Organic Frameworks and Fatty Acids
Source: Molecules. 2024 Nov 8;29(22):5291. doi: 10.3390/molecules29225291 (PMC11596333; doi:10.3390/molecules29225291)
Supplement: Supplementary file 1 [file molecules-29-05291-s001.zip › molecules-3269458-supplementary.pdf]

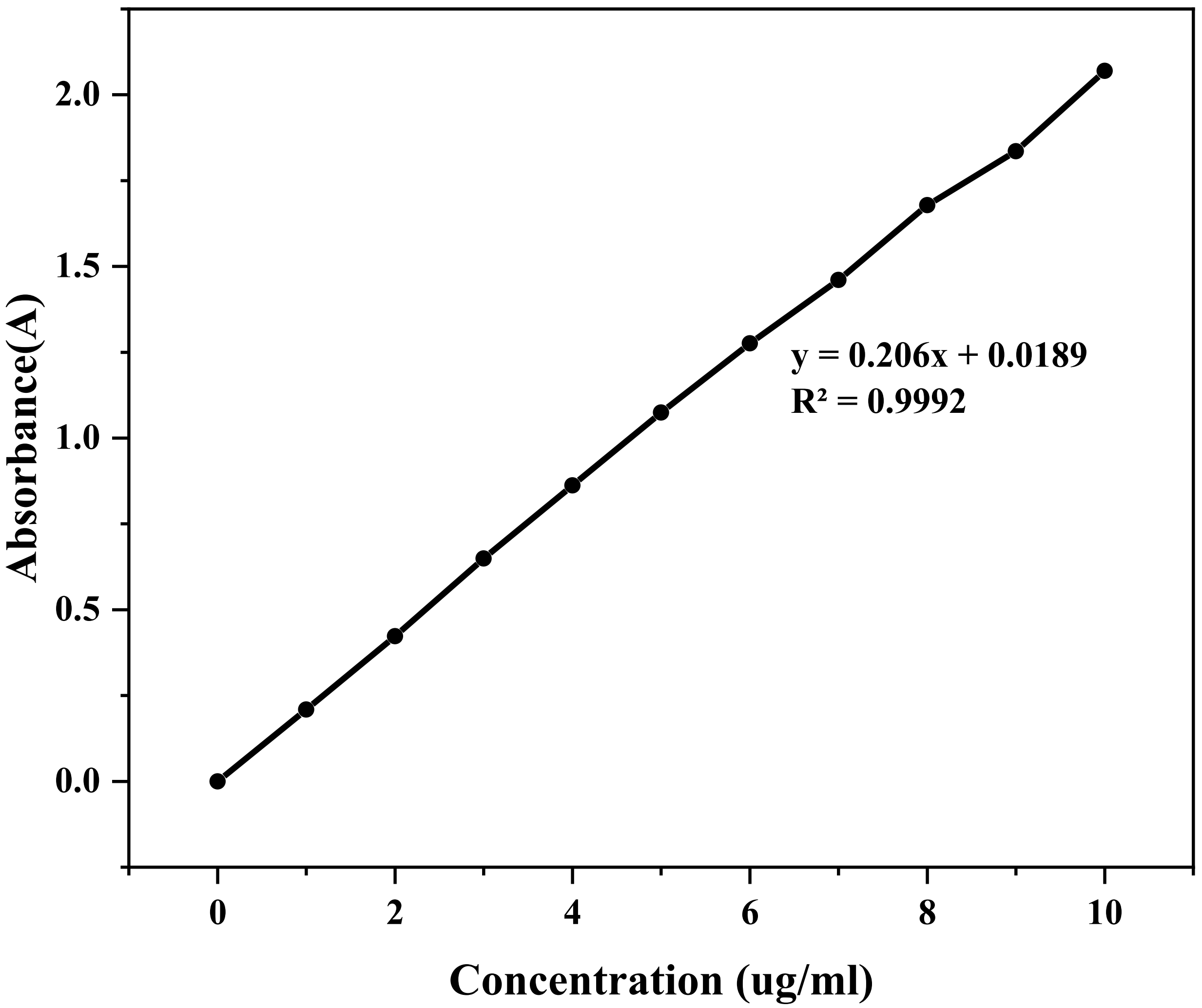

Figure S1. Calibration curve of RhB in phosphate-buffered saline (PBS). The absorbance of RhB at 554 nm was measured at various concentrations to establish a linear relationship between absorbance and concentration. This calibration curve is used to quantify the concentration of RhB released from the drug delivery systems in subsequent experiments. The linear regression equation and R^2 value indicate a high degree of accuracy in the correlation between absorbance and RhB concentration.

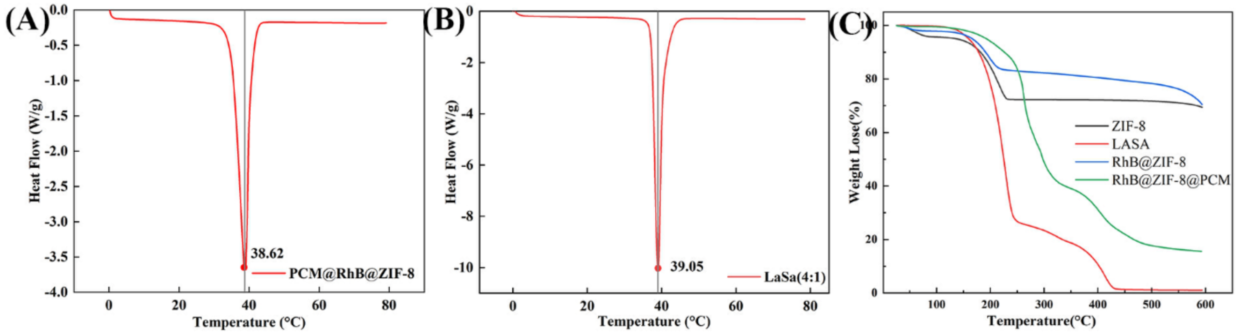

Figure S2. DSC and TGA thermograms of different samples. (A) DSC thermogram of the LA/SA (4:1) eutectic mixture showing a sharp melting peak at 39.05°C. (B) DSC thermogram of RhB@ZIF-8@PCM with a melting peak at 38.62°C, indicating the phase change behavior of the PCM layer. (C) TGA thermograms of ZIF-8, RhB@ZIF-8, LASA, and RhB@ZIF-8@PCM, showing thermal stability and weight loss profiles of each material. The TGA curves reveal that RhB@ZIF-8@PCM exhibits distinct thermal decomposition behavior, corresponding to the degradation of the ZIF-8 framework and the PCM shell.
